# Integrating oxidative-stress biomarkers into a precision oncology risk-stratification model for bladder cancer prognosis and therapy

**DOI:** 10.3389/fcell.2024.1453448

**Published:** 2024-09-16

**Authors:** Jianxu Huang, Dewang Zhou, Weihan Luo, Yujun Liu, Haoxiang Zheng, Yongqiang Wang

**Affiliations:** ^1^ Shantou University Medical College, Shantou University, Shantou, China; ^2^ Department of Experiment & Research, South China Hospital, Medical School, Shenzhen University, Shenzhen, China; ^3^ Kobilka Institute of Innovative Drug Discovery, School of Medicine, The Chinese University of Hong Kong, Shenzhen, Guangdong, China; ^4^ Medical School, Anhui University of Science and Technology, Huainan, China

**Keywords:** oxidative stress, bladder cancer, prognostic model, risk stratification, treatment

## Abstract

**Introduction:**

Bladder cancer is a common malignant tumor with significant heterogeneity, making personalized risk stratification crucial for optimizing treatment and prognosis. This study aimed to develop a prognostic model based on oxidative stress-related genes to guide risk assessment in bladder cancer.

**Methods:**

Differentially expressed oxidative stress-related genes were identified using the GEO database. Functional enrichment and survival analyses were performed on these genes. A risk-scoring model was built and tested for prognostic value and therapeutic response prediction. Expression of key genes was validated by qRT-PCR in samples from two muscle-invasive and two non-muscle-invasive bladder cancer patients.

**Results:**

Several oxidative stress-related genes were identified as significantly associated with survival. The risk-scoring model stratified patients into high- and low-risk groups, accurately predicting prognosis and therapeutic responses. qRT-PCR confirmed the differential expression of key genes in patient samples.

**Discussion:**

The study provides a concise risk stratification model based on oxidative stress-related genes, offering a practical tool for improving personalized treatment in bladder cancer. Further validation is required for broader clinical application.

## Introduction

Bladder cancer is one of the most common malignancies in men worldwide. Overall, bladder cancer accounts for 3% of newly diagnosed cancer cases, in which incidence is ∼3-fold higher in males than females (Roupret et al., 2021; Sung et al., 2021). According to whether cancer cells invade the bladder wall, bladder cancer can be classified into non-muscle-invasive bladder cancer (NMIBC) and muscle-invasive bladder cancer (MIBC) subtypes (Witjes et al., 2021). Although NMIBC has a relatively favorable prognosis, its frequent recurrence necessitates long-term disease monitoring for patients, which is associated with high healthcare costs. MIBC has a much poorer prognosis with 5-year survival ≤50% due to early occult metastatic dissemination (Sung et al., 2021; Witjes et al., 2021; Flaig et al., 2020).

The therapeutic strategies for bladder cancer are also variable according to the subtypes and the patient’s risk level. Based on National Comprehensive Cancer Network guidelines, NMIBC is usually managed through transurethral resection of bladder tumor (TURBT) prior to an intravesical chemotherapy/immunotherapy (Flaig et al., 2020). For patients with MIBC, more aggressive therapies are recommended to reduce disease-specific mortality. These include radical cystectomy, neoadjuvant chemotherapy (NAC), postoperative systemic chemotherapy, and immunotherapy with checkpoint inhibitors. (Patel et al., 2020). Pathologic response after treatment, regardless of types of bladder cancer, is closely associated with recurrence-free survival (RFS) and overall survival (OS) (Rosenblatt et al., 2012). Currently, clinicians are unable to identify which patients will benefit from chemotherapy or immunotherapy. Although somatic deleterious mutations in ERCC2 have been described to correlate with a better response to NAC, some non-responders also have ERCC2 mutations in the same (peri-) helicase regions, suggesting other factors affect NAC sensitivity (Kim et al., 2016; Van Allen et al., 2014). Current classification systems also hinder personalized treatment and management strategies for patients. For example, some patients with occult early metastasis were pathologically classified as low-risk diseases (Piao et al., 2022). As such, there is an urgent need for innovative classification approaches that facilitate accurate diagnosis, individualized treatment, and assessment of the prognosis of bladder cancer.

Oxidative stress refers to the state of imbalance between oxidants and antioxidants in which levels of reactive oxygen species (ROS) exceed the antioxidant defense mechanisms of the cells (Aggarwal et al., 2019). Accumulating studies have revealed oxidative stress involves development of malignant diseases from initiation through promotion and progression, until it acquires a highly malignant, drug resistance and metastatic phenotype (Kang and Yang, 2020; Klaunig, 2018; Zahra et al., 2021). Consistent with previous studies on different hallmarks of cancer, oxidative stress is broadly engaged in cancer biology processes, and it has been suggested that the progression of bladder cancer may be associated with NOX-4 and lipid peroxidation (LPO) products resulting from oxidative stress (Moloney and Cotter, 2018; Shimada et al., 2011). LPO can increase arachidonic acid metabolism, producing malondialdehyde (MDA) due to elevated levels of cyclooxygenase-2 (Wigner et al., 2021). Lepara Z et al. demonstrated that MDA levels correlated with the clinical stages and grade of bladder cancer (Lepara et al., 2020; Pande et al., 2011). Hui Deng et al. reported that lysyl oxidase-like 4 regulate oxidative stress pathway activity to impact on the multi-chemoresistance *in vitro* and *in vivo* (Deng et al., 2014). Overall, multiple aspects of cancer development have been identified as correlated with oxidative stress, but less attention has been paid to applying these genes to predict prognosis and therapeutic efficiency of bladder cancer and re-subgrouping bladder cancer.

In this study, Oxidative stress-related genes (OSRGs) have been identified and validated as practical molecular signatures. Through integrative bioinformatics analysis and qRT-PCR, these signatures can be used to construct a stratified risk prognostic model for bladder cancer. Validation studies have demonstrated the value of this model in assessing prognosis, classifying patients, and predicting responses to treatments, indicating its potential utility in facilitating individualized treatment strategies for bladder cancer.

## Materials and methods

### Data acquisition

A derivation cohort of 408 bladder cancer patient samples, along with their gene expression profiles and clinical data, was sourced from the TCGA public database (https://cancergenome.nih.gov/) for this study. The two independent testing cohorts (GSE13507 and GSE32894) datasets were downloaded from GEO (https://www.ncbi.nlm.nih.gov/geo/). The Series Matrix File data of GSE13507 involves 165 cases of BLCA samples, and the Series Matrix File data of GSE32894 includes 224 bladder cancer patient samples. For the purpose of identifying oxidative stress-related genes, 2,642 genes with a Relevance Score >3 were obtained from GeneCards. (https://www.genecards.org).

### Identification of differentially expressed gene and functional analysis

Data from 165 bladder cancer patients were downloaded from the official website of GEO (https://www.ncbi.nlm.nih.gov/geo/) database. Within these patients, 104 had Ta and T1 samples, while 61 had > T1 samples. We set the fold change >1.5 and *P*-value <0.05 as the inclusion criteria to determine oxidative stress DEGs. Utilizing ClusterProfiler R package, Gene Ontology (GO) and Kyoto Encyclopedia of Genes and Genomes (KEGG) analyses were conducted based on the oxidative stress DEGs. GO and KEGG enrichment pathways with P and Q values less than 0.05 were considered significant categories.

### Model construction and prognosis

The TCGA-BLCA cohort was set as the training cohort to analyze the oxidative stress DEGs using univariate COX regression analysis. A total of 72 prognostic candidates (*p* < 0.05) were identified from the analysis. Further filtering using the least absolute shrinkage and selection operator (LASSO) COX regression analysis identified 21 genes. To enhance the effectiveness and accuracy of prognostic prediction, the prognosis-related DEGs were subjected to a further analysis utilizing multivariate COX regression. This approach yielded a final set of 11 oxidative stress DEGs, based on which the oxidative stress risk score was calculated.

$$\text{Risk score}=\sum \left(\beta i\times \text{Expi}\right)$$

(i refers to the number of screened prognostic oxidative stress-related genes, β refers to the regression coefficient of the gene).

The risk score for each sample was calculated utilizing the specified formula, and the optimal cut-off point for the risk score was identified with the aid of the survminer R package. Patients were classified into high-risk and low-risk subgroups based on the optimal cut points, and Kaplan–Meier analyses were subsequently conducted to compare survival differences between these two subgroups. To evaluate the prognostic value of the risk model, the time-dependent receiver operating characteristic (ROC) curves and corresponding areas under the curve (AUC) values were utilized in both the training and testing cohorts. In addition, univariate and multivariate COX regression analyses were conducted to further validate the prognostic capability of the risk model. The nomogram and calibration plots were constructed based on the important clinicopathological characteristics and the risk scores to help visualize the clinical significance of the risk model.

### Gene set enrichment analysis

The Gene Set Enrichment Analysis software (version 4.3.2) was utilized to perform the functional enrichment analysis between the high-risk and low-risk subgroups. The normalized enrichment score (NES) represented the degrees of over-expression of gene sets in the low-risk subgroups. The FDR q < 0.25 and nominal *P*-value <0.05 were considered statistical differences.

### Drug sensitivity analysis

The pRRophetic R package was utilized to predict the chemotherapy sensitivity of individual samples, and a comparative analysis was conducted on the IC50 values of each specific chemotherapeutic agents for bladder cancer, distinguishing between the high-risk and low-risk subgroups.

### Immune infiltration analysis

Immune infiltration analysis was performed using the CIBERSORT (Cell-type Identification By Estimating Relative Subsets Of RNA Transcripts) tool, an algorithm designed for quantitative analysis of immune cell types within heterogeneous tissue samples based on gene expression data.

### Risk subgroup predicted the therapeutic response of immune checkpoint blockades (ICBs)

ICBs response scores of each sample were calculated using Tumor Immune Dysfunction and Exclusion (TIDE) (http://tide.dfci.harvard.edu/). Based on TCGA datasets, the limma, reshape2, ggplot2, and ggpubr R packages were used to analyze the correlation of the risk score with immune checkpoint expression and visualize the results.

### Bladder cancer subtyping

Partition the patients in the TCGA-BLCA dataset into high-risk and low-risk groups, and map these groups to the molecular subtypes of BLCA defined in TCGA. Use the BLCA subtyping R package to predict the TCGA subtypes for patients in the GEO dataset, assigning each patient a TCGA subtype label. Then the distribution of TCGA subtypes among patients in the high-risk and low-risk groups will be examined.

### Tissue samples and quantitative real-time PCR

Aiming to validate the expression of 11 candidate genes, two MIBC and two NMIBC tissue samples obtained from the South China Hospital of Shenzhen University were collected. RNA extraction and reverse transcription were performed according to the manufacturer’s instructions (Vazyme, RC112-01 and R323-01). Relative qPCR (ΔΔCt method) was performed in triplicate using ABI Prism7000 Sequence Detection System (Applied Biosystems). Gene expression levels were normalized to GAPDH transcript levels (Applied Biosystems). Primers of 11 genes were listed in Supplementary Table S1.

### Statistical analysis

Several R packages, including limma, survival, survminer, glmnet, and timeROC, were used to perform our data analysis. The empirical Bayesian approach of the limma R package was utilized to identify oxidative stress-related DEGs. The survminer package, widely used for survival analysis visualization in R, offers the surv_cutpoint function, which finds the optimal threshold based on the maximum selected rank statistics method and visualizes the results. Survival curves were generated by the Kaplan-Meier method and compared using the log-rank test. The R language (version 4.2.1) was used for all statistical studies. All statistical tests were two-sided, and we considered a *p*-value less than 0.05 as a statistically significant difference.

## Results

### Identification of candidate oxidative stress-related genes in bladder cancer

**FIGURE 1:**
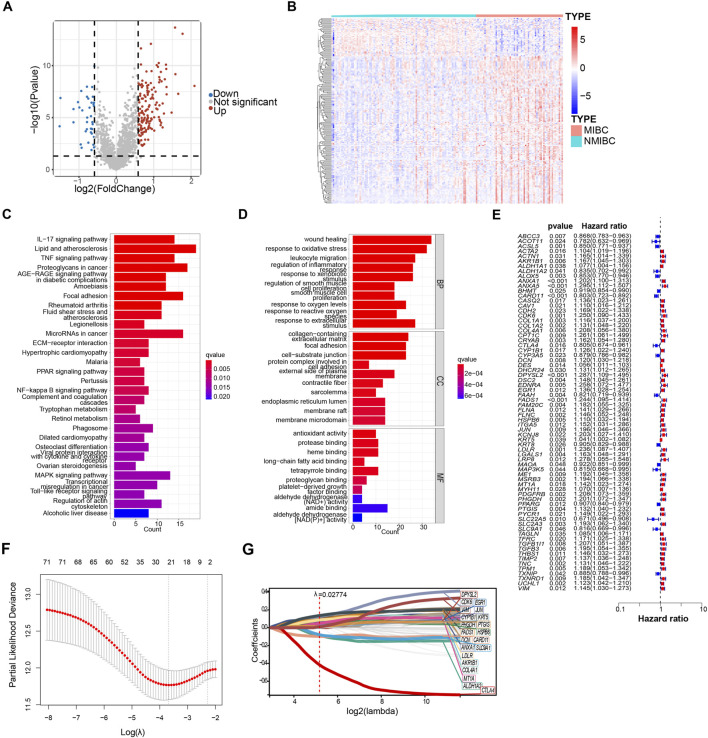
Identification of oxidative stress-related genes in model construction. **(A)** Differentially expressed oxidative stress-related genes of GSE13507 between non–muscle-invasive bladder cancer (NMIBC) and muscle-invasive bladder cancer (MIBC) were shown in volcano plot. The blue and red dots represented DEGs filtered based on the cutoff criteria of |fold change|≥1.5 and *P*-value <0.05. The grey dots represented genes that do not satisfy the cutoff criteria. **(B)** The heatmap showed the oxidative stress-related DEGs. **(C, D)** The oxidative stress-related DEGs were analyzed by Kyoto Encyclopedia of Genes and Genomes (KEGG) and gene ontology (GO). Significantly enriched pathways **(C)** and terms **(D)** were shown. **(E)** Forest plot of 72 oxidative stress-related DEGs that related to overall survival via univariate Cox regression analyses. **(F)** The parameter was screened by LASSO regression. **(G)** LASSO regression of 72 oxidative stress-related DEGs.

To investigate which OSRGs may be involved in bladder cancer development, Gene expression profiles were extracted from the GSE13507 bladder cancer cohort, comprising 104 NMIBC samples and 61 MIBC samples. In the subsequent step, a comparison was conducted between the NMIBC and MIBC samples, which led to the identification of differentially expressed genes (DEGs) that had a fold change magnitude of 1.5 or greater and a *P*-value lower than 0.05. Ultimately, 184 candidate genes were obtained through the overlap of DEGs and OSRGs for subsequent analysis. The expression of differentially oxidative stress-related genes was visualized via the heatmap and the volcano plots (Figures 1A, B). Among 184 candidate genes, 146 genes were upregulated and 38 genes were downregulated (Figures 1A, B).

In order to delve deeper into the potential functions of the candidate genes, a total of 184 DEGs were subjected to the functional enrichment analysis. The top 30 significant enriched gene ontology (GO) and KEGG pathways are shown in bar charts (Figures 1C, D). Except for oxidative stress, gene ontology biological process (GO-BP) revealed that DEGs were mainly related to wound healing and regulation of inflammatory response. Wound healing and tumorigenesis are two processes that rely on similar molecular mechanisms. Tumor formation is characterized by the continuous activation of wound healing associated pathways involved (Arwert et al., 2012). Similar to the finding from GO enrichment analysis, the results from KEGG enrichment analysis indicated that DEGs were mainly associated with MAPK signaling pathway and inflammatory response, such as IL−17 signaling pathways, TNF signaling pathways, and toll-like receptor signaling pathways. The mitogen-activated protein kinase (MAPK) pathways are crucial regulators of the cellular processes that fuel the malignant transformation of normal cells and cancer progression (Sinkala et al., 2021).

In the endeavor to establish a prognostic model, we applied the univariate COX regression analysis to identify a total of 72 survival-related oxidative stress-related DEGs based on 403 bladder cancer samples from the TCGA database, and results were shown in the forest plot (Figure 1E). Of these, 55 genes with HRs >1, indicating risk genes, while the remaining 17 genes had HRs <1, suggesting protection genes (Figure 1E). 21 genes were further identified using the least absolute shrinkage and selection operator (LASSO) COX regression analysis with λ = 0.02774709 (Figures 1F, G). Finally, to enhance the effectiveness and accuracy of prognostic prediction, we applied multivariate COX regression to yield a final set of 11 oxidative stress-related DEGs. Among 11 oxidative stress-related genes, *AKR1B1, CDK6, CYP1B1, EGR1, HSPB6, LDLR, MT1A*, and *PHGDH* were identified as risk genes, while *ALDH1A2, CARD11*, and *CTLA4* were identified as protective genes.

#### A prognostic model was constructed and validated based on selected oxidative stress-associated genes

Based on selected 11 oxidative stress-related DEGs, the risk score was calculated. The risk score formula was as follows:

$$\text{Risk score}=\left(0.1653\right)*AKR1B1+\left(-0.2276\right)*ALDH1A2+\left(-0.1243\right)*CARD11+\left(0.2525\right)*CDK6+\left(-0.6258\right)*CTLA4+\left(0.1737\right)*CYP1B1+\left(0.1097\right)*EGR1+\left(0.0666\right)*HSPB6+\left(0.1844\right)*LDLR+\left(0.1056\right)*MT1A+\left(0.2065\right)*PHGDH$$

**FIGURE 2:**
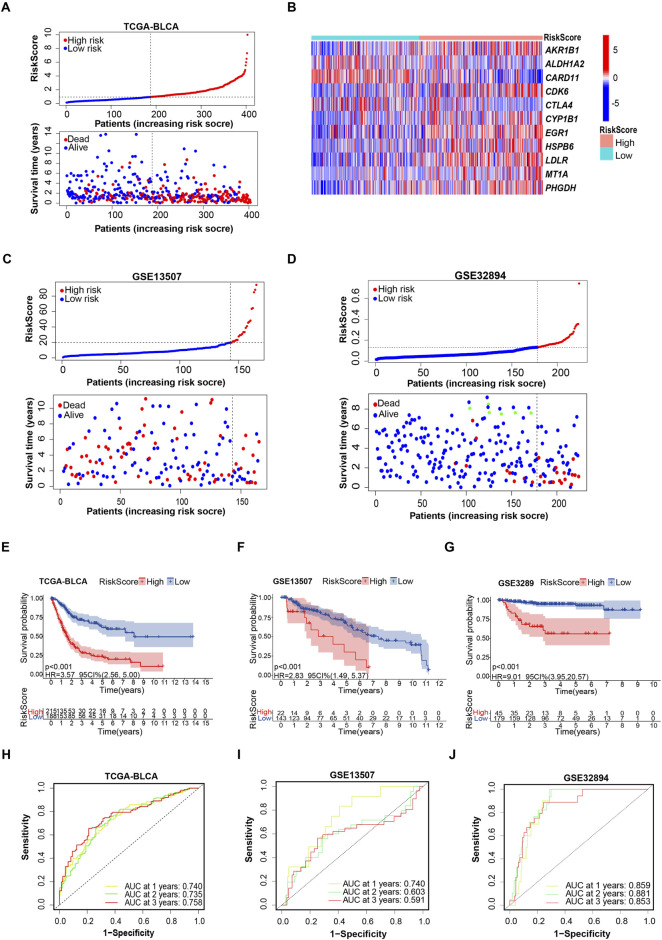
Evaluating the capacity of the prognostic model in survival prediction. **(A, B)** Risk plot distribution, survival status of patients, and heat map of expression of included genes in the training cohort. **(C, D)** Risk plot distribution and survival status of patients in the testing cohort. **(E–G)** Kaplan–Meier survival curves for the prognostic risk model. **(H–J)** The time-dependent ROC curves of the prognostic risk model.

Next, the patients in both the training cohorts and the testing cohorts were stratified into high-risk and low-risk subgroups by the optimal cut point of risk score. We found that following the risk plot distribution and survival status of patients, patients in the high-risk subgroup had significantly higher mortality rates than those in the low-risk subgroup (Figures 2A–D). For the expression of selected OSRGs, the heatmap showed that the expressions of *AKR1B1, CDK6, CYP1B1, EGR1, HSPB6, LDLR, MT1A, and PHGDH* were higher in the high-risk subgroup, while the expressions of *ALDH1A2, CARD11*, and *CTLA4* were lower in the high-risk subgroup (Figure 2B).

A deeper exploration of the credibility of risk score-based subgrouping was conducted through the analysis of time-dependent receiver operating characteristic (ROC) curves and their corresponding areas under the curve (AUC) values in both training and testing cohorts, validating the grouping based on the prognostic model is reliable. Kaplan–Meier survival analyses indicated that the patients in the high-risk subgroup had shorter survival time than those in the low-risk subgroup in both the training and testing cohorts (Figures 2E, F). The 1-, 3-, and 5-year survival probability of the risk score was represented by the AUC values of 0.740, 0.735, and 0.758, respectively, in the TCGA training cohort (Figure 2H). Consistent with the results in the training cohort, we also obtained satisfactory results in testing cohorts. Particularly, the testing cohort (GSE13507) exhibited AUC values of 0.740, 0.603, and 0.591 at the 1-year, 2-year, and 3-year marks, respectively. (Figure 2I). The 1-, 2-, and 3-year AUC values of ROC curves for the prognostic risk model in another testing cohort (GSE32894) were 0.859, 0.881, and 0.853, respectively (Figure 2J). These findings established a feasible prognostic model, based on selected 11 oxidative stress associated genes.

### Independent prognostic analysis and matching with TCGA subtypes

**FIGURE 3:**
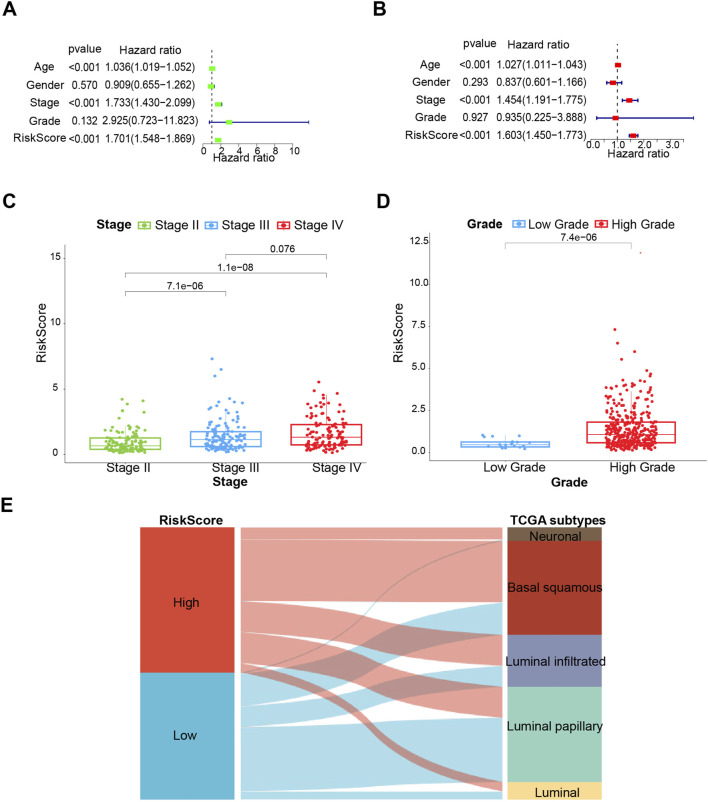
The clinical prognostic value of the prognostic risk model. **(A)** Univariate analysis. **(B)** Multivariate analysis. **(C, D)** Associations between the risk score and tumor stage and grade. **(E)** Sankey diagram of subtype distributions in groups with different risk scores. *p < 0.05, **p < 0.01, ***p < 0.001.

Upon further integration of risk scores with age and clinical characteristics, both univariate and multivariate Cox regression analyses were conducted. Age, stage, and risk score were revealed to be independent prognostic factors (Figures 3A, B). According to the statistical principle, the small sample size of stage I patients in the TCGA-BLCA cohort makes it infeasible to compare with other stages. Hence, comparisons were limited to stages II, III, and IV, and the risk score was found to be positively correlated with stage and grade (Figures 3C, D). We also assigned our low-risk and high-risk subgroups with TCGA subtypes of bladder cancer. The Sankey diagram showed that the high-risk group mainly matched with more malignant phenotypes like luminal_infiltrated, basal_squamous and neuronal, while the low-risk group is prone to matching with less malignant phenotypes, such as luminal_papilary (Figure 3E). Consistently, the other two cohorts (GSE32894 and GSE13507) supported Figure 3E results well (Supplementary Figures S1A, B).

### The establishment of a comprehensive nomogram

**FIGURE 4:**
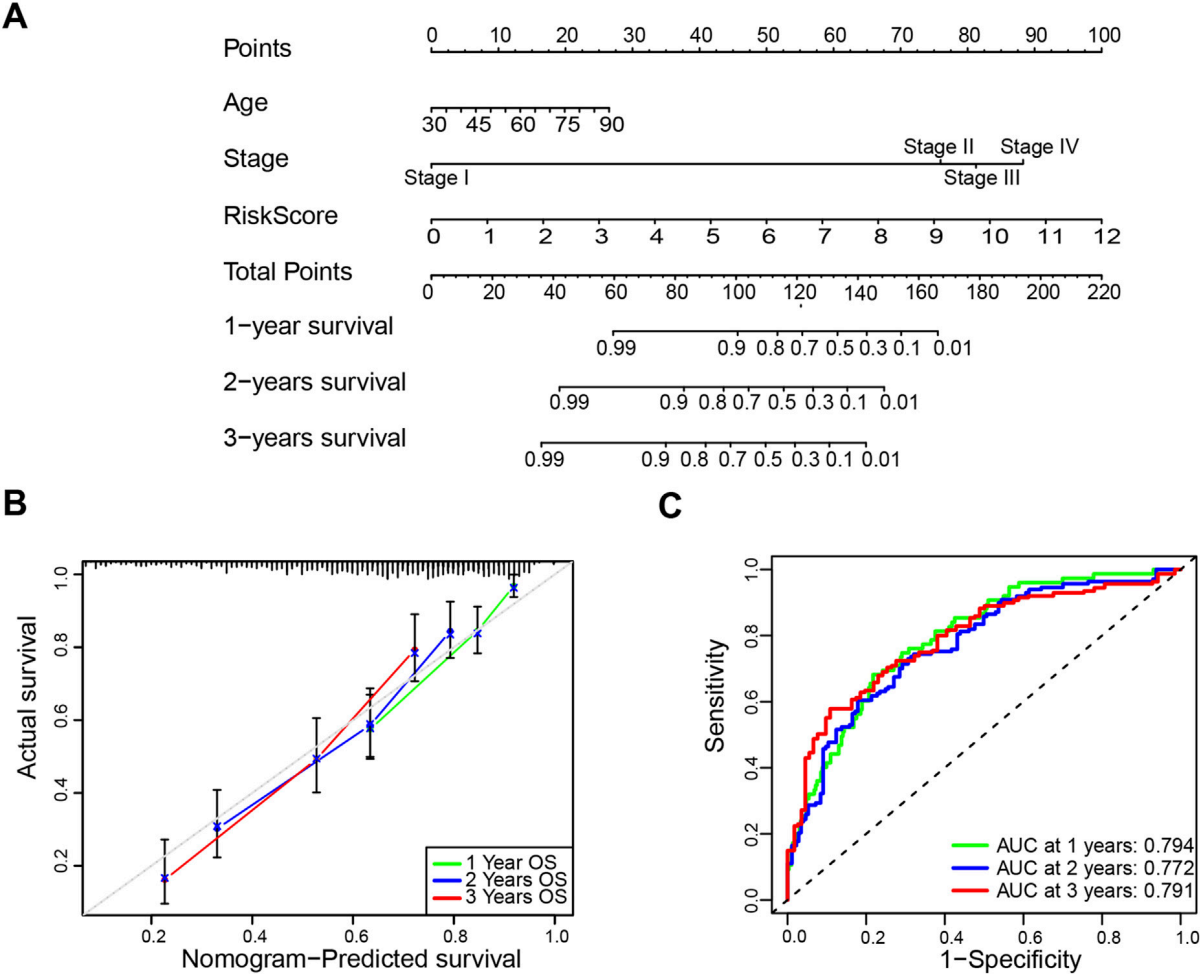
Construction and validation of the nomogram. **(A)** The nomogram based on age, stage and risk score to predict the 1-year, 3-year, and 5-year overall survival. **(B)** Calibration plots for the overall survival nomogram model. **(C)** Receiver operating characteristic (ROC) curves for the nomogram based on the TCGA-BLCA cohort.

Next, a comprehensive nomogram was developed based on the age, stage, and risk score of bladder cancer patients (Figure 4A). The calibration curves indicated a significant consistency between nomogram predictions and actual observations (Figure 4B). In addition, the 1-, 2-, and 3-year AUC values of ROC curves for the nomogram in the TCGA cohort were 0.794, 0.772, and 0.791, respectively (Figure 4C). To evaluate the predictive power of the prognostic risk model for prognosis in multiple bladder cancer subgroups, stratification survival analysis was conducted based on age (<60 years and ≥60 years), gender (Male and Female), and stage (Stage I ∼ II and Stage III ∼ IV). The Kaplan–Meier survival analyses showed that the high-risk patients had significantly shorter overall survival compared to the low-risk patients in all subtypes (*p* < 0.01; Supplementary Figures S2A–F). Collectively, these results suggest that the prognostic model is credible, and it has an independent predictive value for the prognosis and subgrouping of bladder cancer.

### Gene enrichment analysis based on high- and low-risk groups

Further validation of the credibility of the high- and low-risk subgrouping was achieved through GSEA, which was performed based on DEGs identified between the two groups. The results showed that immune-response associated process and pathway were both enriched in the low-risk group, suggesting that patients in the low-risk group may benefit more from immunotherapy (Supplementary Figures S3A, C). The GO BP enrichment results indicated that cell growth, negative regulation of apoptotic signaling pathway, regulation of cellular response to growth factor stimulus, and negative regulation of oxidative stress-induced cell death were significantly enriched in the high-risk group (Supplementary Figure S3B). The KEGG pathway enrichment revealed significant enrichment of cell cycle, WNT-signaling, oocyte meiosis, and focal adhesion in the high-risk group (Supplementary Figure S3D).Taken together, the high-risk group exhibits more malignant characteristics, further suggesting subgrouping-based risk score is reliable.

### Treatment sensitivity analysis between high- and low-risk groups

**FIGURE 5:**
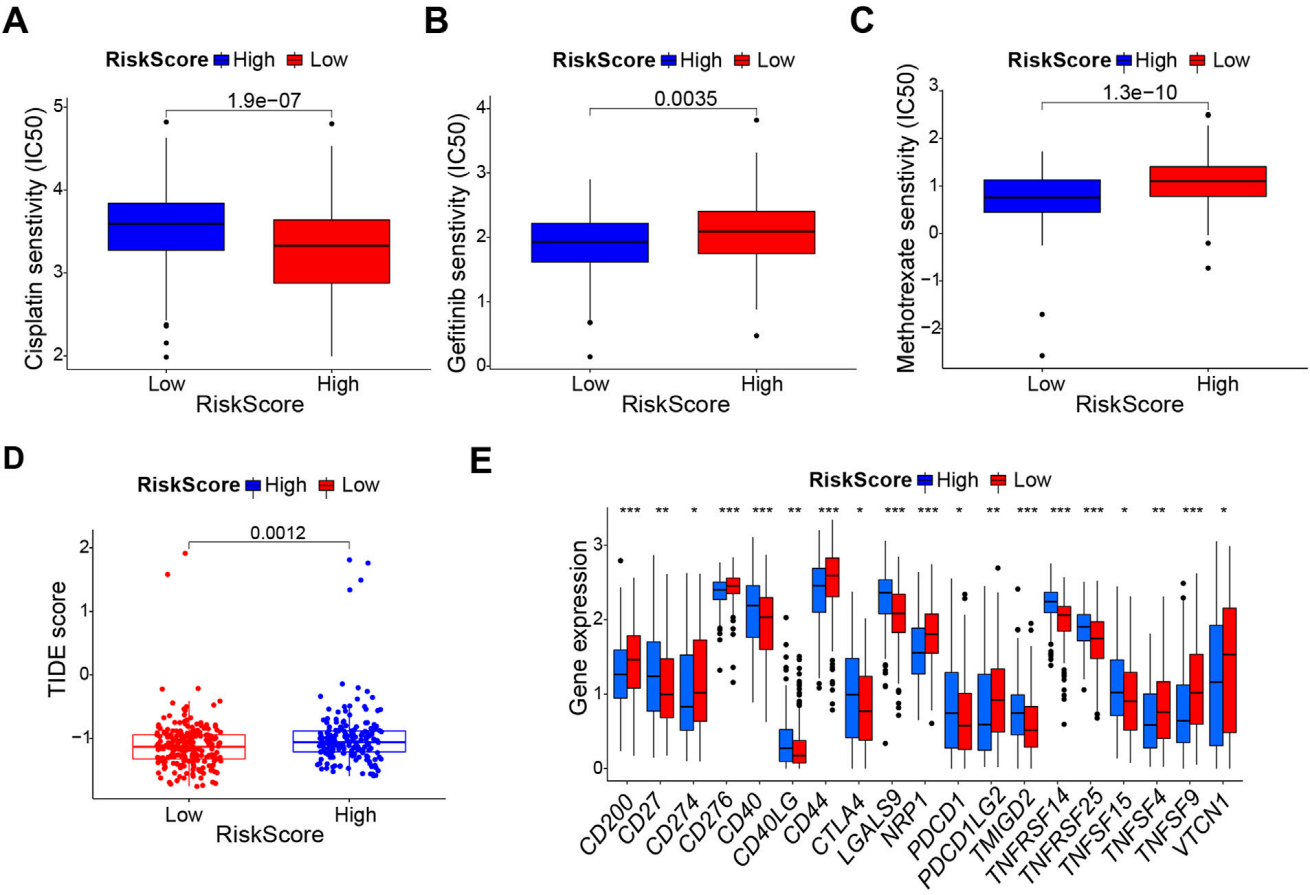
The prognostic model provides a potential guideline for therapeutic strategies of bladder cancer. **(A–C)** Comparison of sensitivity to Cisplatin, Gefitinib, and Methotrexate in different risk score group. **(D)** Comparison of ICB response scores. **(E)** Comparison of immune checkpoint genes between high - and low-risk groups. *p < 0.05, **p < 0.01, ***p < 0.001.

To explore the chemotherapy sensitivity in high- and low-risk groups, IC50 was utilized as a measure to evaluate chemosensitivity. The analysis revealed that there was a significant decrease in IC50 of cisplatin in the high-risk groups, suggesting patients in the high-risk group were more sensitive to cisplatin, consistent with findings in the basal_squamous subtype (Figure 5A). However, the IC50 of the high-risk group was higher than the low-risk group for Gefitinib and Methotrexate (Figures 5B, C).

Functional enrichment analysis has shown OSRGs are associated with immune response (Figures 1C, D). Therefore, we further examined the difference in the efficacy of immunotherapy between high- and low-risk groups through the ICB response scoring (TIDE score). Lower ICB response scores were revealed in the high-risk subgroup compared to the low-risk subgroup (Figure 5D). To further investigate the relationships between immune checkpoints and the risk score, expression levels of 19 immune checkpoint genes were calculated, including *CD274, CD200, CD27, CD276, CD40, CD40LG, CD44, CTLA4, LGALS9, NRP1, PDCD1, PDCD1LG2, TMIGD2, TNFRSF14, TNFRSF25, TNFSF15, TNFSF4, TNFSF9, and VTCN1.* We observed that the expression levels of the *CD200, CD274, CD276, CD44, NRP1, PDCD1LG2, TNFSF4, TNFSF9,* and *VTCN1* were higher in the high-risk group than in the low-risk group, while the expression of *CD27, CD40, CD40LG, CTLA4, LGALS9, PDCD1, TMIGD2, TNFRSF14, TNFRSF25*, and *TNFSF15* were lower in the high-risk group (Figure 5E).

### Validation of 11 candidate genes

**FIGURE 6:**
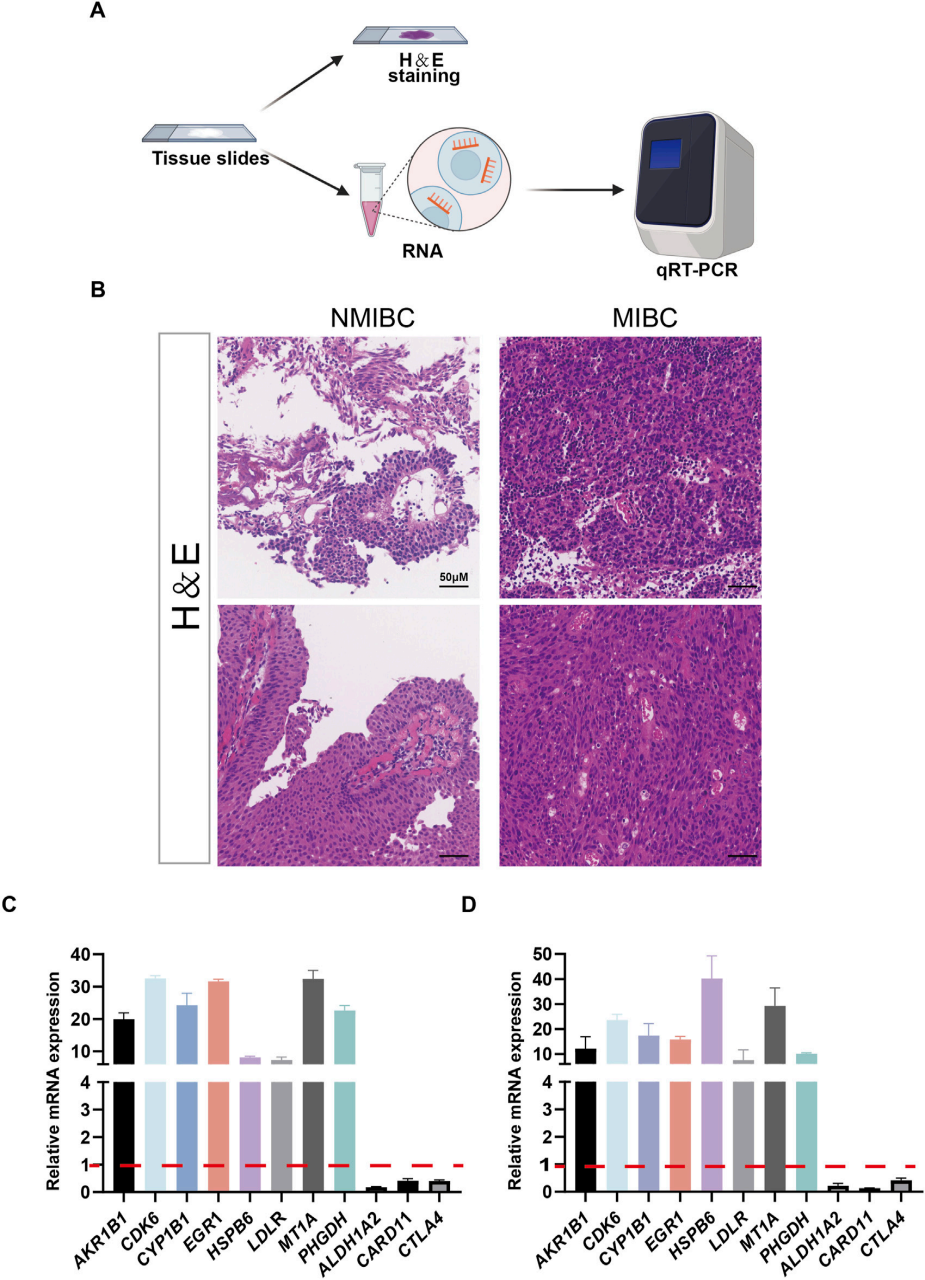
The expressions of 11 selected genes were validated by quantitative real-time PCR (qRT-PCR). **(A)** Schematic representation of the H&E stainning and RNA extraction of patient samples. **(B)** Results of H&E stained bladder cancer samples from two patients. **(C, D)** Expression of 11 genes at the mRNA level detected by qRT-PCR.

NMIBC and MIBC tissues were employed as experimental materials to validate the expression patterns of the 11 candidate genes in bladder cancer. As shown in Figure 6A, tumor tissue slides were firstly subjected to H&E staining to identify tumor phenotypes, following that RNA was extracted from tissue slides for qRT-PCR (Figure 6A). Hematoxylin and Eosin staining (H&E staining) confirmed the successful collection of 2 NMIBC and 2 MIBC samples (Figure 6B). The results of qRT-PCR showed that *AKR1B1, CDK6, CYP1B1, EGR1, HSPB6, LDLR, MT1A,* and *PHGDH* were upregulated in MIBC samples, while *ALDH1A2, CARD11*, and *CTLA4* were downregulated in MIBC samples (Figures 6C, D).

## Discussion

Current stratification methods for bladder cancer may not fully account for the genetic and molecular diversity of the disease, leading to patients within the same stage potentially exhibiting vastly different biological behaviors and treatment responses. In this study, 11 oxidative stress-related genes were selected to establish a credible prognostic model with stratified risk for bladder cancer, revealing that risk score is an independent prognosis factor. The proposed model not only achieves classification of patients into high- and low-risk subgroups, exhibiting concordance with TCGA subtypes, but furthermore, it serves as an effective tool for clinicians in assessing the potential responsiveness of patients to cisplatin-based chemotherapy or immunotherapy, relying on their risk scores.

Oxidative stress involves development of malignant diseases from initiation through promotion and progression, until it acquires a highly malignant, drug resistance and metastatic phenotype (Aggarwal et al., 2019; Klaunig, 2018; Zahra et al., 2021; Moloney and Cotter, 2018). Despite the significance of oxidative stress in bladder cancer progression, OSRG screening for prognosis prediction remains insufficiently investigated. Advances in sequencing and bioinformatics facilitate a enhanced selection of candidate genes, improving prognostic accuracy. As shown in screened eleven oxidative stress-related DEGs, *AKR1B1* is necessary for tumor growth (Li et al., 2022) and was found to interact with signal transducer and activator of transcription 3 (STAT3) (Zhang et al., 2021), participating in anti-cell death processes and leading to drug resistance. Acting at the interface of *p53* and *RB*, *CDK6* contributes to tumor initiation by promoting cell cycle and antagonizing stress responses (Bellutti et al., 2018). *CYP1B1* upregulates in the advanced stages of bladder cancer and participates in the activation of procarcinogen (Al-Saraireh et al., 2021; Salinas-Sanchez et al., 2012). *EGR1* has been shown to regulate genes influencing proliferation, apoptosis, immune cell activation, and matrix degradation, among others (Li et al., 2019). Metallothioneins (MTs) are small cysteine-rich proteins that play significant roles in tumor formation, progression, and drug resistance, with *MT1A* being one of the functional isoforms (Si and Lang, 2018). *PHGDH* is amplified in the malignant tumor and is essential for nucleotide production and cell proliferation in highly aggressive brain metastatic cells (Ngo et al., 2020). In addition, *ALDH1A2, CARD11* and *CTLA4* are implicated in cancer immunity and can be useful prognostic biomarkers in some cancer types (Liu et al., 2020; Carter and Pomerantz, 2022; Zhu et al., 2022). In this study, the 11 genes mentioned above were supported well from clinical samples (Figures 6C, D).

Although current treatment strategies have prolonged the overall survival of bladder cancer patients, the absolute improvement of overall survival was limited in a subgroup of those who received chemotherapy/immunotherapy (Galsky et al., 2016; Jiang et al., 2019; Kamat et al., 2017; Tran et al., 2021). These non-responders may suffer from increased modality, making this standard care of bladder cancer poorly applied (Galsky et al., 2011). Through our prognostic model, if patients are identified as high risk, clinicians can choose Cisplatin as the candidate drug, rather than choosing immunotherapy and Gefitinib and Methotrexate. Based on the immune infiltration results of tumor samples (Supplementary Figure S4), patients in the low-risk group exhibit higher infiltration of CD8^+^ T cells. CD8^+^ T cells are crucial anti-tumor immune cells capable of directly killing tumor cells, which partly explains the better prognosis in the low-risk group. Cisplatin can induce immunogenic cell death in tumor cells. This process releases tumor antigens and damage-associated molecular patterns (DAMPs), which activate dendritic cells and subsequently activate and recruit T cells, particularly CD8^+^ T cells, thereby enhancing the anti-tumor immune response. In contrast, the high-risk group shows insufficient CD8^+^ T cell infiltration in the tumor microenvironment. The application of cisplatin can improve the infiltration of CD8^+^ T cells in these patients.

Despite the achievements in this study, there are still some limitations that should be acknowledged. Firstly, relatively small sample size of bladder cancer patients limits the reliability and generalizability of our prognostic model, requiring larger datasets for future validation. Secondly, while the clinical value of the risk model has been validated across multiple public cohorts, further prospective clinical trials and molecular mechanism investigations are crucial to confirm its clinical significance and elucidate the underlying mechanisms. Finally, the significant heterogeneity of bladder cancer poses a challenge to the model’s universal applicability, as molecular diversity and clinical presentation variability of tumors may lead to inconsistencies in model predictions, thereby limiting its comprehensiveness. To address these limitations and enhance model’s predictive accuracy, future research endeavors should aspire to integrate a more comprehensive set of biomarkers and clinical data into the model.

## Conclusion

In conclusion, in this study, a new risk-stratification model based on 11 oxidative stress-related genes has been successfully developed. The risk score proved to be a reliable independent risk factor for predicting overall survival, subgrouping patients and closely associated with the clinical characteristics of bladder cancer. Additionally, the findings provide valuable insights into personalized treatment approaches for bladder cancer patients, particularly by predicting the correlation between immunotherapy or chemotherapy response and risk score. Finally, the expression of 11 candidate genes has been validated by patient samples of NMIBC and MIBC.

## Data Availability

The original contributions presented in the study are included in the article/Supplementary Material, further inquiries can be directed to the corresponding author.

## Supplementary material

SUPPLEMENTARY TABLE S1 Primers of 11 oxidative stress-related genes.

SUPPLEMENTARY FIGURE S1 Subtype distributions in groups with different risk scores. **(A, B)** Sankey diagram of subtype distributions in groups with different risk scores in GSE13507 cohort **(A)** and GSE32894 cohort **(B)**, respectively.

SUPPLEMENTARY FIGURE S2 Kaplan–Meier survival curves for the different prognostic factors. **(A)** age<60 **(B)** age>60 **(C)** Male **(D)** Female **(E)** Stage Ⅰ∼Ⅱ **(F)** Stage Ⅲ∼Ⅳ.

SUPPLEMENTARY FIGURE S3 Gene set enrichment analysis of risk model. **(A)** Gene set enrichment analysis of GOBP immunology memory process and GOBP positive regulation of T cell receptors signaling pathway in low-risk samples versus high-risk samples. **(B)** Gene set enrichment analysis of GOBP cell growth, GOBP negative regulation of apoptotic signaling pathway, GOBP negative regulation of oxidative stress induced death, and GOBP regulation of cellular response to growth factor stimulus in low-risk samples versus high-risk samples. **(C)** Gene set enrichment analysis of KEGG antigen processing and presentation, KEGG primary immunodeficiency, and KEGG RIG_I like receptor signaling pathway in low-risk samples versus high-risk samples. **(D)** Gene set enrichment analysis of KEGG cell cycle, KEGG focal adhesion, KEGG oocyte meiosis, and KEGG WNT signaling pathway in low-risk samples versus high-risk samples.

SUPPLEMENTARY FIGURE S4 The immune infiltration results of tumor samples. *p < 0.05, **p < 0.01, ***p < 0.001.

## Abbreviations

NMIBC, non-muscle-invasive bladder cancer; MIBC, muscle-invasive bladder cancer; TURBT, transurethral resection of bladder tumour; NAC, neoadjuvant chemotherapy; RFS, recurrence-free survival; OS, overall survival; ROS, reactive oxygen species; LPO, lipid peroxidation; MDA, malondialdehyde; OSRGs, oxidative stress-related genes; DEGs, differentially expression genes; GO-BP, gene ontology biological process; MAPK, mitogen-activated protein kinase; ROC, receiver operating characteristic; AUC, corresponding areas under the curve; KEGG, Kyoto Encyclopedia of Genes and Genomes; ICB, immune checkpoint-blocking therapy; LASSO, least absolute shrinkage and selection operator; TIDE, tumor immune dysfunction and exclusion; GSEA, gene set enrichment analysis; H&E staining, Hematoxylin and Eosin staining.

## References

[B1] Aggarwal V. Tuli H. S. Varol A. Thakral F. Yerer M. B. Sak K. (2019). Role of reactive oxygen species in cancer progression: molecular mechanisms and recent advancements. Biomolecules 9 (11), 735. 10.3390/biom9110735 31766246 PMC6920770

[B2] Al-Saraireh Y. M. Alshammari F. Youssef A. M. M. Al-Sarayreh S. Almuhaisen G. H. Alnawaiseh N. (2021). Profiling of CYP4Z1 and CYP1B1 expression in bladder cancers. Sci. Rep. 11 (1), 5581. 10.1038/s41598-021-85188-4 33692504 PMC7946900

[B3] Arwert E. N. Hoste E. Watt F. M. (2012). Epithelial stem cells, wound healing and cancer. Nat. Rev. Cancer 12 (3), 170–180. 10.1038/nrc3217 22362215

[B4] Bellutti F. Tigan A. S. Nebenfuehr S. Dolezal M. Zojer M. Grausenburger R. (2018). CDK6 antagonizes p53-induced responses during tumorigenesis. Cancer Discov. 8 (7), 884–897. 10.1158/2159-8290.CD-17-0912 29899063 PMC6031305

[B5] Carter N. M. Pomerantz J. L. (2022). CARD11 signaling in regulatory T cell development and function. Adv. Biol. Regul. 84, 100890. 10.1016/j.jbior.2022.100890 35255409 PMC9149070

[B6] Deng H. Lv L. Li Y. Zhang C. Meng F. Pu Y. (2014). miR-193a-3p regulates the multi-drug resistance of bladder cancer by targeting the LOXL4 gene and the oxidative stress pathway. Mol. Cancer 13, 234. 10.1186/1476-4598-13-234 25311867 PMC4200202

[B7] Flaig T. W. Spiess P. E. Agarwal N. Bangs R. Boorjian S. A. Buyyounouski M. K. (2020). Bladder cancer, version 3.2020, NCCN clinical practice guidelines in oncology. J. Natl. Compr. Canc Netw. 18 (3), 329–354. 10.6004/jnccn.2020.0011 32135513

[B8] Galsky M. D. Hahn N. M. Rosenberg J. Sonpavde G. Hutson T. Oh W. K. (2011). Treatment of patients with metastatic urothelial cancer “unfit” for Cisplatin-based chemotherapy. J. Clin. Oncol. 29 (17), 2432–2438. 10.1200/JCO.2011.34.8433 21555688

[B9] Galsky M. D. Stensland K. D. Moshier E. Sfakianos J. P. McBride R. B. Tsao C. K. (2016). Effectiveness of adjuvant chemotherapy for locally advanced bladder cancer. J. Clin. Oncol. 34 (8), 825–832. 10.1200/JCO.2015.64.1076 26786930

[B10] Jiang D. M. Jiang H. Chung P. W. M. Zlotta A. R. Fleshner N. E. Bristow R. G. (2019). Neoadjuvant chemotherapy before bladder-sparing chemoradiotherapy in patients with nonmetastatic muscle-invasive bladder cancer. Clin. Genitourin. Cancer 17 (1), 38–45. 10.1016/j.clgc.2018.09.021 30686350

[B11] Kamat A. M. Bellmunt J. Galsky M. D. Konety B. R. Lamm D. L. Langham D. (2017). Society for Immunotherapy of Cancer consensus statement on immunotherapy for the treatment of bladder carcinoma. J. Immunother. Cancer 5 (1), 68. 10.1186/s40425-017-0271-0 28807024 PMC5557323

[B12] Kang Q. Yang C. (2020). Oxidative stress and diabetic retinopathy: molecular mechanisms, pathogenetic role and therapeutic implications. Redox Biol. 37, 101799. 10.1016/j.redox.2020.101799 33248932 PMC7767789

[B13] Kim J. Mouw K. W. Polak P. Braunstein L. Z. Kamburov A. Kwiatkowski D. J. (2016). Somatic ERCC2 mutations are associated with a distinct genomic signature in urothelial tumors. Nat. Genet. 48 (6), 600–606. 10.1038/ng.3557 27111033 PMC4936490

[B14] Klaunig J. E. (2018). Oxidative stress and cancer. Curr. Pharm. Des. 24 (40), 4771–4778. 10.2174/1381612825666190215121712 30767733

[B15] Lepara Z. Lepara O. Fajkic A. Rebic D. Alic J. Spahovic H. (2020). Serum malondialdehyde (MDA) level as a potential biomarker of cancer progression for patients with bladder cancer. Rom. J. Intern Med. 58 (3), 146–152. 10.2478/rjim-2020-0008 32364521

[B16] Li L. Ameri A. H. Wang S. Jansson K. H. Casey O. M. Yang Q. (2019). EGR1 regulates angiogenic and osteoclastogenic factors in prostate cancer and promotes metastasis. Oncogene 38 (35), 6241–6255. 10.1038/s41388-019-0873-8 31312026 PMC6715537

[B17] Li Q. Wang R. Yang Z. Li W. Yang J. Wang Z. (2022). Molecular profiling of human non-small cell lung cancer by single-cell RNA-seq. Genome Med. 14 (1), 87. 10.1186/s13073-022-01089-9 35962452 PMC9375433

[B18] Liu J. N. Kong X. S. Huang T. Wang R. Li W. Chen Q. F. (2020). Clinical implications of aberrant PD-1 and CTLA4 expression for cancer immunity and prognosis: a pan-cancer study. Front. Immunol. 11, 2048. 10.3389/fimmu.2020.02048 33072070 PMC7539667

[B19] Moloney J. N. Cotter T. G. (2018). ROS signalling in the biology of cancer. Semin. Cell Dev. Biol. 80, 50–64. 10.1016/j.semcdb.2017.05.023 28587975

[B20] Ngo B. Kim E. Osorio-Vasquez V. Doll S. Bustraan S. Liang R. J. (2020). Limited environmental serine and Glycine confer brain metastasis sensitivity to PHGDH inhibition. Cancer Discov. 10 (9), 1352–1373. 10.1158/2159-8290.CD-19-1228 32571778 PMC7483776

[B21] Pande D. Negi R. Khanna S. Khanna R. Khanna H. D. (2011). Vascular endothelial growth factor levels in relation to oxidative damage and antioxidant status in patients with breast cancer. J. Breast Cancer 14 (3), 181–184. 10.4048/jbc.2011.14.3.181 22031798 PMC3200512

[B22] Patel V. G. Oh W. K. Galsky M. D. (2020). Treatment of muscle-invasive and advanced bladder cancer in 2020. CA Cancer J. Clin. 70 (5), 404–423. 10.3322/caac.21631 32767764

[B23] Piao X. M. Kim S. K. Byun Y. J. Zheng C. M. Kang H. W. Kim W. T. (2022). Utility of a molecular signature for predicting recurrence and progression in non-muscle-invasive bladder cancer patients: comparison with the EORTC, CUETO and 2021 EAU risk groups. Int. J. Mol. Sci. 23 (22), 14481. 10.3390/ijms232214481 36430959 PMC9696895

[B24] Rosenblatt R. Sherif A. Rintala E. Wahlqvist R. Ullén A. Nilsson S. (2012). Pathologic downstaging is a surrogate marker for efficacy and increased survival following neoadjuvant chemotherapy and radical cystectomy for muscle-invasive urothelial bladder cancer. Eur. Urol. 61 (6), 1229–1238. 10.1016/j.eururo.2011.12.010 22189383

[B25] Roupret M. Babjuk M. Burger M. Capoun O. Cohen D. Compérat E. M. (2021). European association of urology guidelines on upper urinary tract urothelial carcinoma: 2020 update. Eur. Urol. 79 (1), 62–79. 10.1016/j.eururo.2020.05.042 32593530

[B26] Salinas-Sanchez A. S. Donate-Moreno M. J. Lopez-Garrido M. P. Gimenez-Bachs J. M. Escribano J. (2012). Role of CYP1B1 gene polymorphisms in bladder cancer susceptibility. J. Urol. 187 (2), 700–706. 10.1016/j.juro.2011.10.063 22177211

[B27] Shimada K. Fujii T. Anai S. Fujimoto K. Konishi N. (2011). ROS generation via NOX4 and its utility in the cytological diagnosis of urothelial carcinoma of the urinary bladder. BMC Urol. 11, 22. 10.1186/1471-2490-11-22 22032647 PMC3215170

[B28] Si M. Lang J. (2018). The roles of metallothioneins in carcinogenesis. J. Hematol. Oncol. 11 (1), 107. 10.1186/s13045-018-0645-x 30139373 PMC6108115

[B29] Sinkala M. Nkhoma P. Mulder N. Martin D. P. (2021). Integrated molecular characterisation of the MAPK pathways in human cancers reveals pharmacologically vulnerable mutations and gene dependencies. Commun. Biol. 4 (1), 9. 10.1038/s42003-020-01552-6 33398072 PMC7782843

[B30] Sung H. Ferlay J. Siegel R. L. Laversanne M. Soerjomataram I. Jemal A. (2021). Global cancer statistics 2020: GLOBOCAN estimates of incidence and mortality worldwide for 36 cancers in 185 countries. CA Cancer J. Clin. 71 (3), 209–249. 10.3322/caac.21660 33538338

[B31] Tran L. Xiao J. F. Agarwal N. Duex J. E. Theodorescu D. (2021). Advances in bladder cancer biology and therapy. Nat. Rev. Cancer 21 (2), 104–121. 10.1038/s41568-020-00313-1 33268841 PMC10112195

[B32] Van Allen E. M. Mouw K. W. Kim P. Iyer G. Wagle N. Al-Ahmadie H. (2014). Somatic ERCC2 mutations correlate with cisplatin sensitivity in muscle-invasive urothelial carcinoma. Cancer Discov. 4 (10), 1140–1153. 10.1158/2159-8290.CD-14-0623 25096233 PMC4238969

[B33] Wigner P. Grebowski R. Bijak M. Saluk-Bijak J. Szemraj J. (2021). The interplay between oxidative stress, inflammation and angiogenesis in bladder cancer development. Int. J. Mol. Sci. 22 (9), 4483. 10.3390/ijms22094483 33923108 PMC8123426

[B34] Witjes J. A. Bruins H. M. Cathomas R. Compérat E. M. Cowan N. C. Gakis G. (2021). European association of urology guidelines on muscle-invasive and metastatic bladder cancer: summary of the 2020 guidelines. Eur. Urol. 79 (1), 82–104. 10.1016/j.eururo.2020.03.055 32360052

[B35] Zahra K. F. Lefter R. Ali A. Abdellah E. C. Trus C. Ciobica A. (2021). The involvement of the oxidative stress status in cancer pathology: a double view on the role of the antioxidants. Oxid. Med. Cell Longev. 2021, 9965916. 10.1155/2021/9965916 34394838 PMC8360750

[B36] Zhang K. R. Zhang Y. F. Lei H. M. Tang Y. B. Ma C. S. Lv Q. M. (2021). Targeting AKR1B1 inhibits glutathione *de novo* synthesis to overcome acquired resistance to EGFR-targeted therapy in lung cancer. Sci. Transl. Med. 13 (614), eabg6428. 10.1126/scitranslmed.abg6428 34613810

[B37] Zhu L. Kamalathevan P. Koneva L. A. Zarebska J. M. Chanalaris A. Ismail H. (2022). Variants in ALDH1A2 reveal an anti-inflammatory role for retinoic acid and a new class of disease-modifying drugs in osteoarthritis. Sci. Transl. Med. 14 (676), eabm4054. 10.1126/scitranslmed.abm4054 36542696

