# Supplementary figures and images for: Integrating oxidative-stress biomarkers into a precision oncology risk-stratification model for bladder cancer prognosis and therapy
Source: Front Cell Dev Biol. 2024 Sep 16;12:1453448. doi: 10.3389/fcell.2024.1453448 (PMC11439827; doi:10.3389/fcell.2024.1453448)

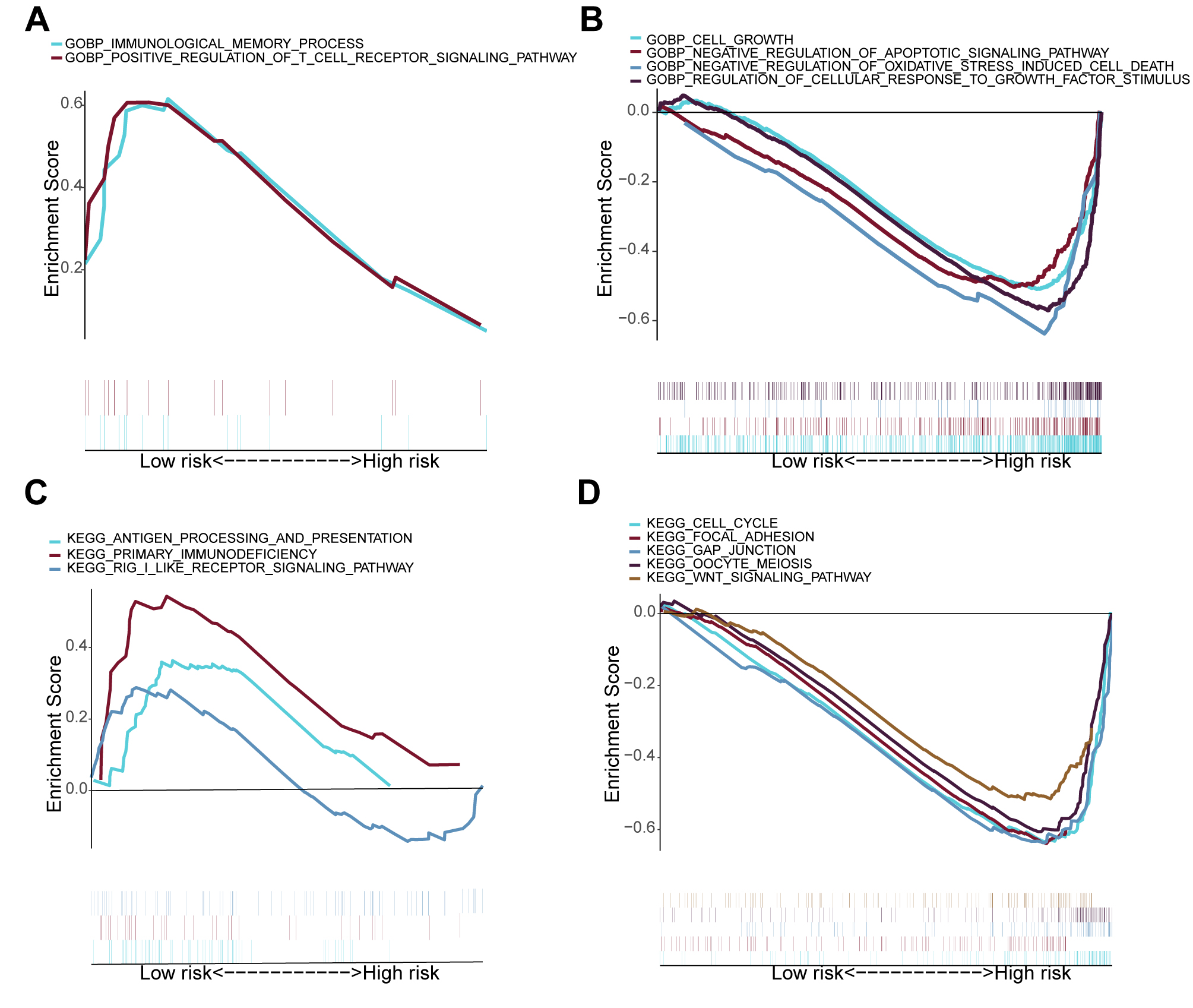

Supplement: Supplementary file 1 [file Image3.JPEG]

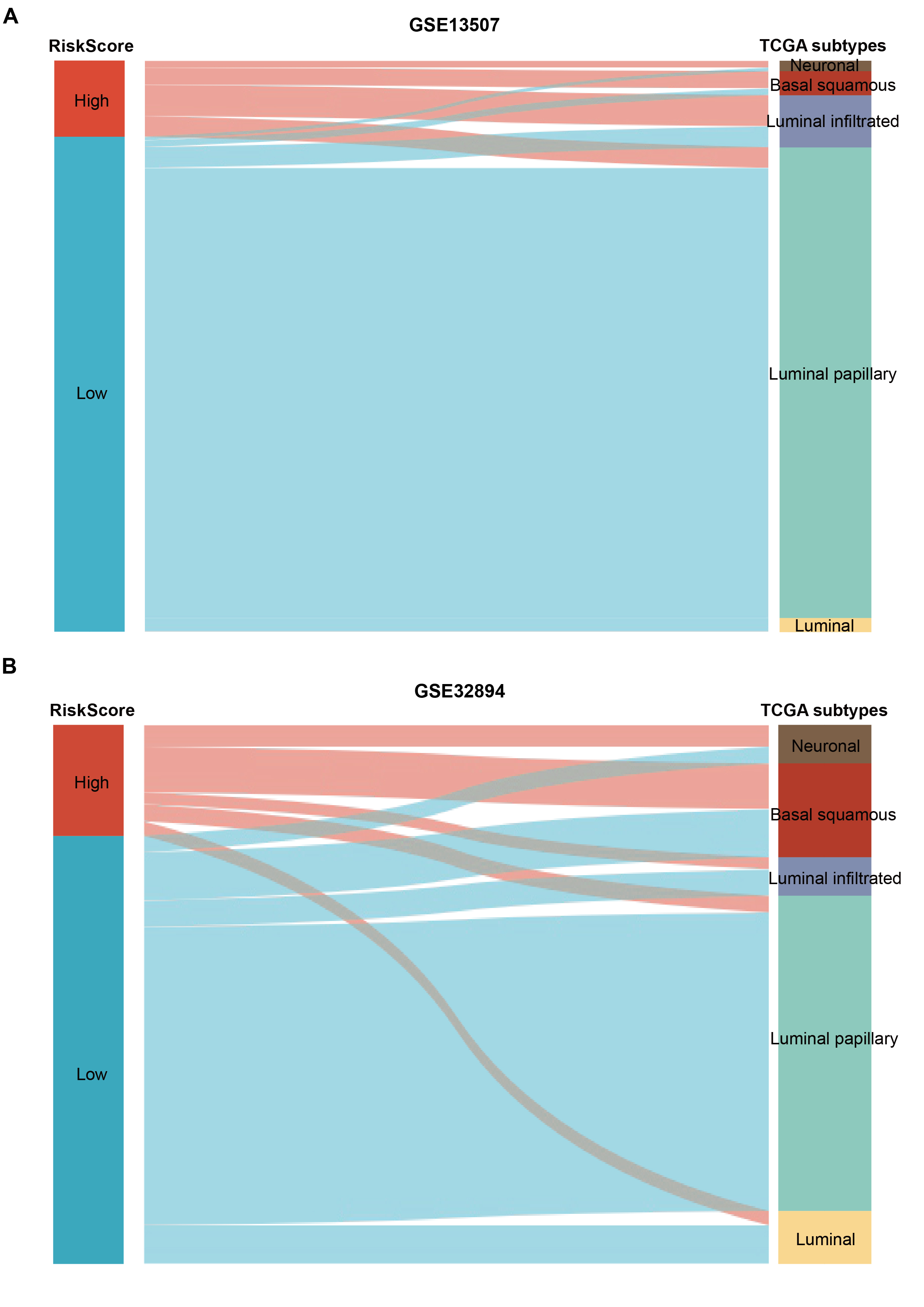

Supplement: Supplementary file 3 [file Image1.JPEG]

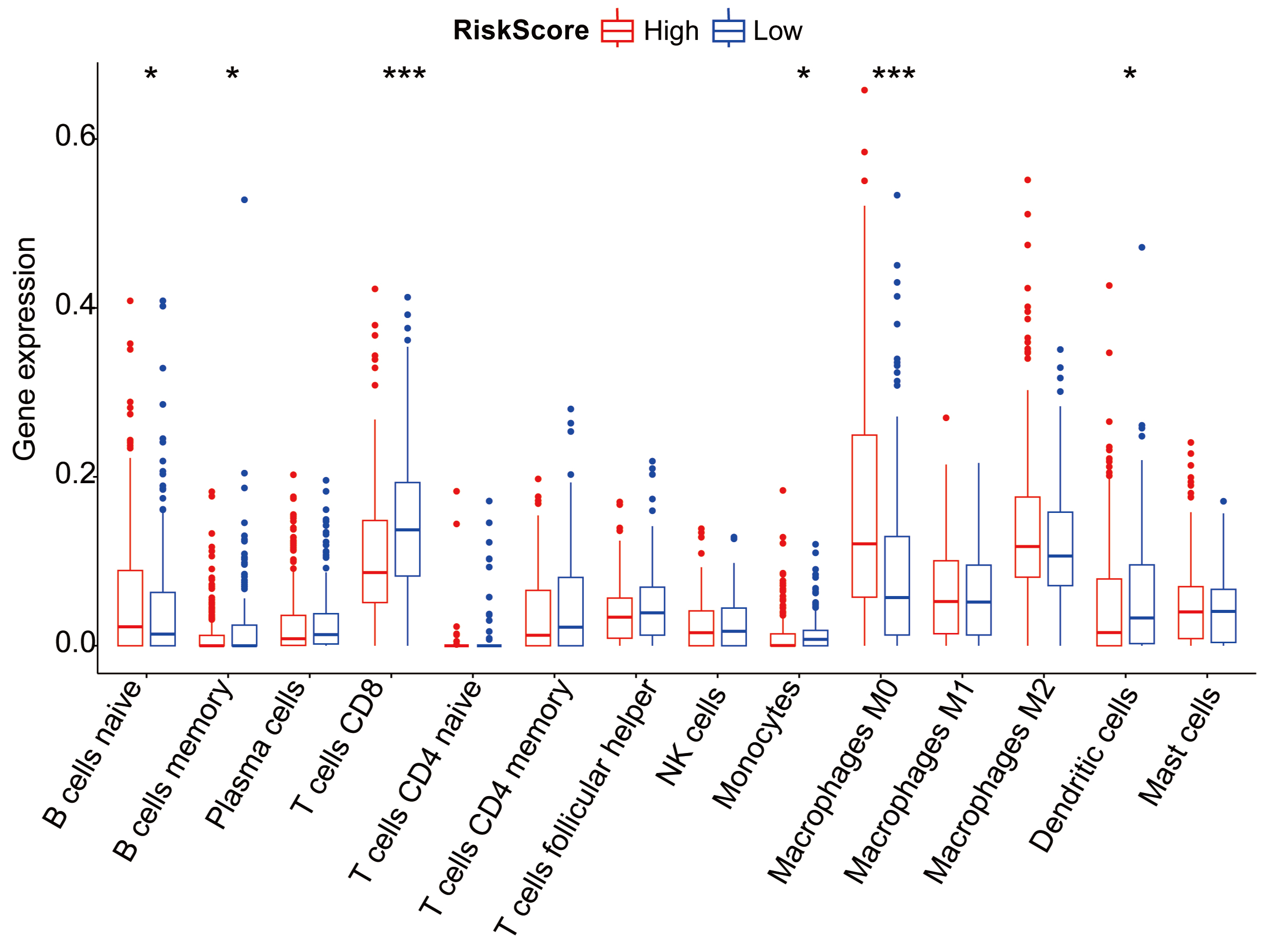

Supplement: Supplementary file 4 [file Image4.JPEG]

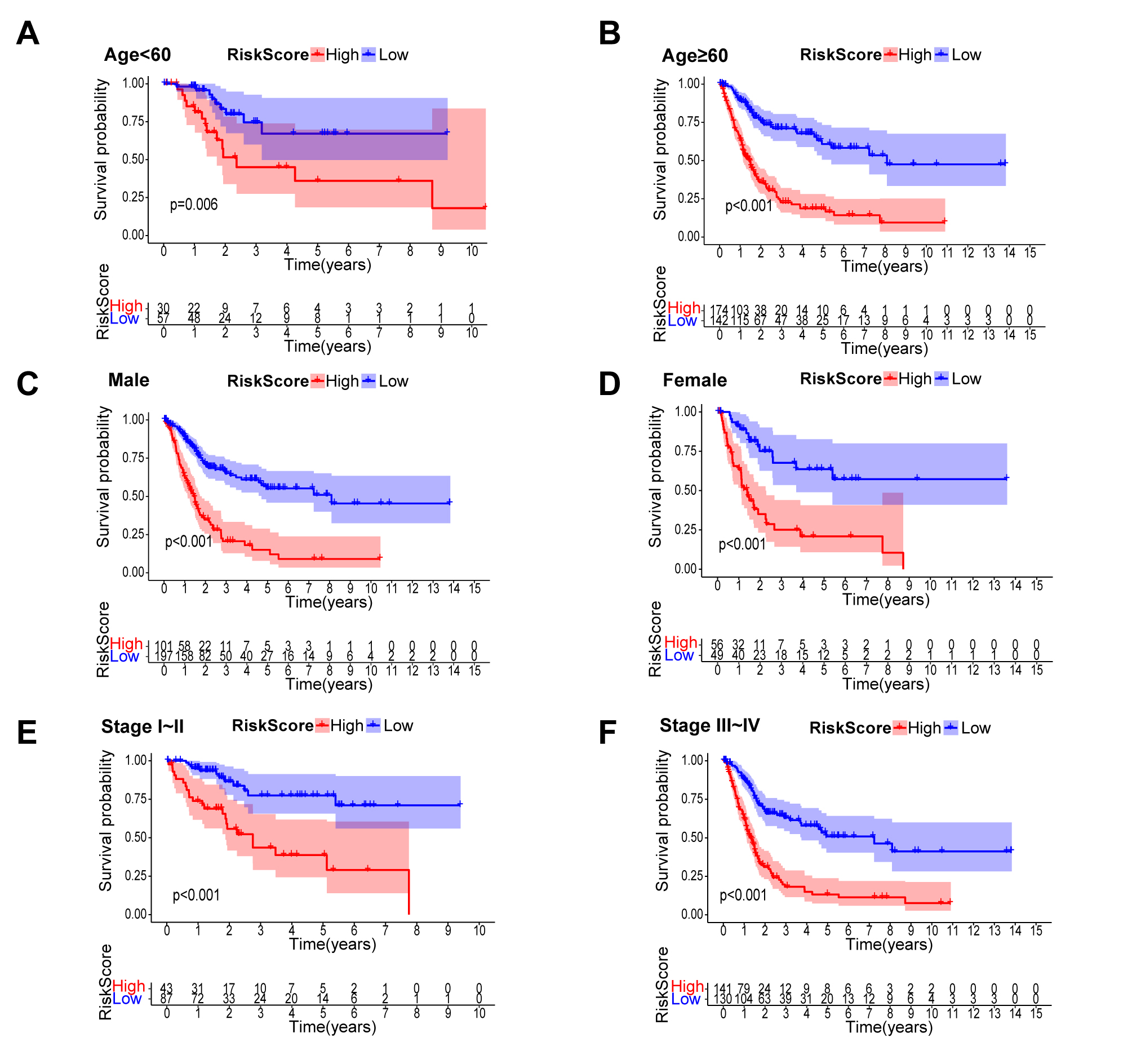

Supplement: Supplementary file 5 [file Image2.JPEG]
