# Supplementary material for: Integrating oxidative-stress biomarkers into a precision oncology risk-stratification model for bladder cancer prognosis and therapy
Source: Front Cell Dev Biol. 2024 Sep 16;12:1453448. doi: 10.3389/fcell.2024.1453448 (PMC11439827; doi:10.3389/fcell.2024.1453448)
Supplement: Supplementary file 2 [file Table1.DOCX]

| **Name of Genes** | **Sequences** |
| --- | --- |
| *AKR1B1* | Forward: AGCCAGGATATGACCACCTTAC |
|  | Reverse: CTTGTGGGAGGTACAGCTCAA |
| *CDK6* | Forward: CCAGATGGCTCTAACCTCAGT |
|  | Reverse: AACTTCCACGAAAAAGAGGCTT |
| *CYP1B1* | Forward: TGAGTGCCGTGTGTTTCGG |
|  | Reverse: GTTGCTGAAGTTGCGGTTGAG |
| *EGR1* | Forward: GGTCAGTGGCCTAGTGAGC |
|  | Reverse: GTGCCGCTGAGTAAATGGGA |
| *HSPB6* | Forward: TGCTAGACGTGAAGCACTTCT |
|  | Reverse: ACCACCTTGACAGCAATTTCC |
| *LDLR* | Forward: ACCAACGAATGCTTGGACAAC |
|  | Reverse: ACAGGCACTCGTAGCCGAT |
| *MT1A* | Forward: CTGGCTCCTGCAAATGCAAA |
|  | Reverse: GCACTTCTCTGATGCCCCTT |
| *PHGDH* | Forward: CTGCGGAAAGTGCTCATCAGT |
|  | Reverse: TGGCAGAGCGAACAATAAGGC |
| *ALDH1A2* | Forward: AGTGTTTTCCAACGTCACTGAT |
|  | Reverse: AGTCTGAGTTATTGGCTCTTTCG |
| *CARD11* | Forward: GGACGCCTTGTGGGAGAATG |
|  | Reverse: TCAATGACCTTACACTGACGC |
| *CTLA4* | Forward: CATGATGGGGAATGAGTTGACC |
|  | Reverse: TCAGTCCTTGGATAGTGAGGTTC |
